# Supplementary material for: Using step selection functions to analyse human mobility using telemetry data in infectious disease epidemiology: a case study of leptospirosis
Source: medRxiv. 2025 Sep 8:2025.04.28.25326582. Originally published 2025 May 1. Preprint. [Version 3] doi: 10.1101/2025.04.28.25326582 (PMC12060945; doi:10.1101/2025.04.28.25326582)

medRxiv preprint doi: https://doi.org/10.1101/2025.04.28.25326582; this version posted September 8, 2025. The copyright holder for this preprint (which was not certified by peer review) is the author/funder, who has granted medRxiv a license to display the preprint in perpetuity.
It is made available under a CC-BY 4.0 International license .

Supplementary Material I

Descriptive Statistics

Telemetry data

The mean number of hours of telemetry data provided by an individual was 13.3 hours, with a standard deviation of 13.5 hours. The mean number of locations recorded by the GPS loggers was 2767 points (SD = 1947.2). There were no differences in the number of hours or number of locations recorded by gender, age or leptospirosis antibody status. There were notable differences in the number of hours recorded and the number of locations by study area. Study area 1 (NVS) had the lowest number of hours recorded (mean = 5.6 hours, SD = 5.6), whilst all other areas had similar hours recorded (area 2: mean = 15.0, SD = 11.4; area 3: mean = 10.9, SD = 14.0; area 4; mean = 20.7, SD = 15.3). The mean number of locations recorded were all similar across all study areas (area 1: mean = 2048, SD = 1206; area 2: mean = 2831, SD = 1302; area 3: mean = 2992, SD = 2737; area 4: mean = 3107, SD = 1761).

medRxiv preprint doi: https://doi.org/10.1101/2025.04.28.25326582; this version posted September 8, 2025. The copyright holder for this preprint (which was not certified by peer review) is the author/funder, who has granted medRxiv a license to display the preprint in perpetuity.
It is made available under a CC-BY 4.0 International license .

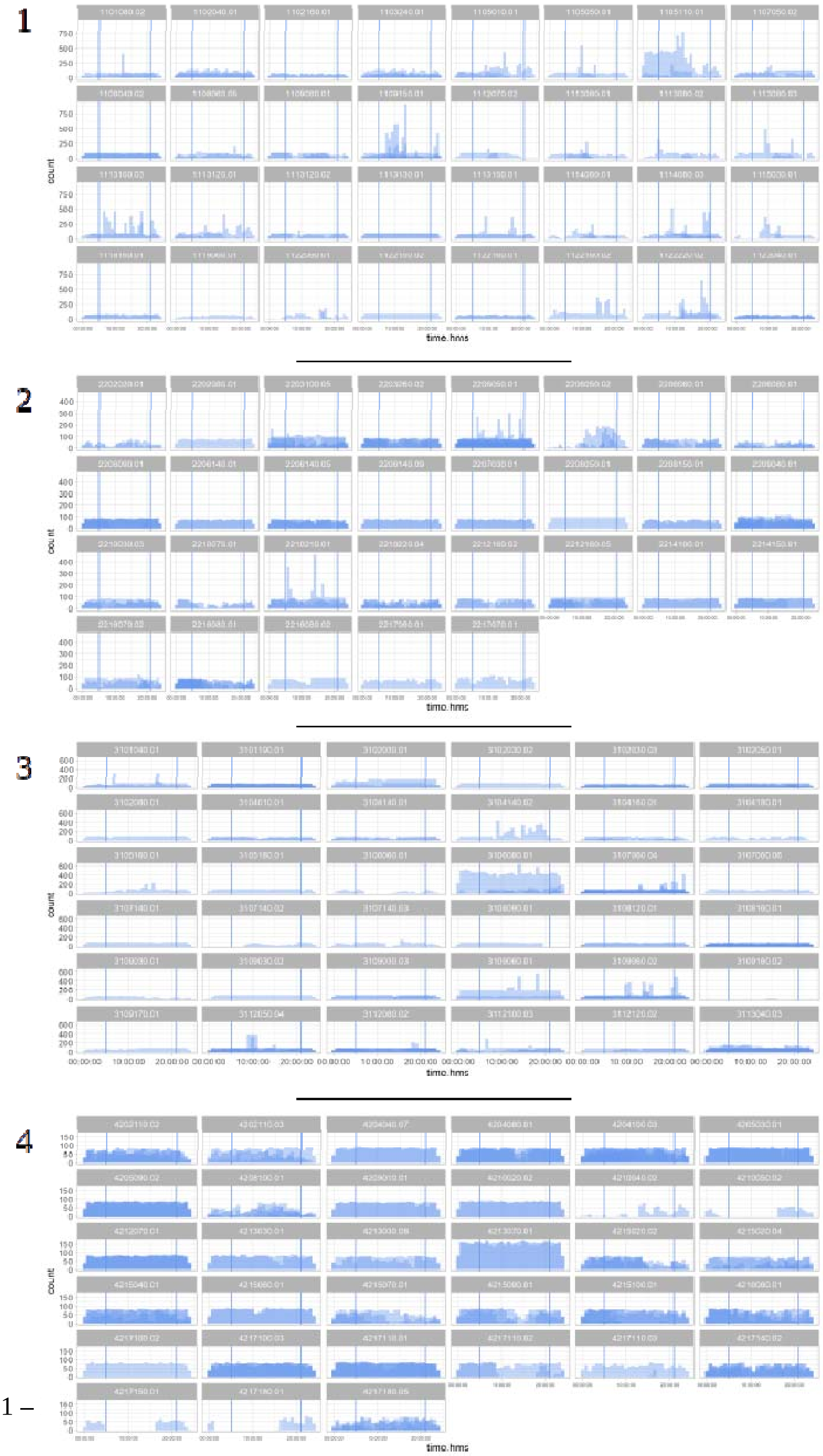

Figure 1 – [illegible] eriods (x

medRxiv preprint doi: https://doi.org/10.1101/2025.04.28.25326582; this version posted September 8, 2025. The copyright holder for this preprint (which was not certified by peer review) is the author/funder, who has granted medRxiv a license to display the preprint in perpetuity.
It is made available under a CC-BY 4.0 International license .

axis), separated into each of the four study areas. Overlapping areas represent multiple days. Vertical bars represent 5 am (left hand bar) and 9 pm (right hand bar), the period of analysis.

Excluded individuals

Table 1 – Demographic details of excluded individuals due to having less than 50 relocations

ID (anonymised)	Relocation below 50	Period	Gender	Age group	Leptospirosis serological status
60	TRUE	05-21	Male	50-54	Neg
91	TRUE	05-21	Male	>55	Neg
15	TRUE	05-09	Female	45-49	Neg
60	TRUE	05-09	Male	50-54	Neg
81	TRUE	05-09	Female	50-54	Neg
91	TRUE	05-09	Male	>55	Neg
108	TRUE	05-09	Female	50-54	Neg
109	TRUE	05-09	Male	20-24	Neg
128	TRUE	05-09	Male	25-29	Neg
129	TRUE	05-09	Male	40-44	Pos
15	TRUE	09-13	Female	45-49	Neg
24	TRUE	09-13	Female	50-54	Neg
60	TRUE	09-13	Male	50-54	Neg
70	TRUE	09-13	Male	35-39	Neg
71	TRUE	09-13	Female	35-39	Neg
76	TRUE	09-13	Male	35-39	Neg
91	TRUE	09-13	Male	>55	Neg

medRxiv preprint doi: https://doi.org/10.1101/2025.04.28.25326582; this version posted September 8, 2025. The copyright holder for this preprint (which was not certified by peer review) is the author/funder, who has granted medRxiv a license to display the preprint in perpetuity.
It is made available under a CC-BY 4.0 International license .

108	TRUE	09-13	Female	50-54	Neg
109	TRUE	09-13	Male	20-24	Neg
128	TRUE	09-13	Male	25-29	Neg
129	TRUE	09-13	Male	40-44	Pos
24	TRUE	13-17	Female	50-54	Neg
60	TRUE	13-17	Male	50-54	Neg
71	TRUE	13-17	Female	35-39	Neg
76	TRUE	13-17	Male	35-39	Neg
91	TRUE	13-17	Male	>55	Neg
5	TRUE	17-21	Male	35-39	Neg
7	TRUE	17-21	Male	50-54	Neg
18	TRUE	17-21	Male	30-34	Neg
22	TRUE	17-21	Male	45-49	Neg
24	TRUE	17-21	Female	50-54	Neg
27	TRUE	17-21	Male	30-34	Neg
30	TRUE	17-21	Female	>55	Neg
60	TRUE	17-21	Male	50-54	Neg
71	TRUE	17-21	Female	35-39	Neg
91	TRUE	17-21	Male	>55	Neg
114	TRUE	17-21	Female	40-44	Neg

Serological data

medRxiv preprint doi: https://doi.org/10.1101/2025.04.28.25326582; this version posted September 8, 2025. The copyright holder for this preprint (which was not certified by peer review) is the author/funder, who has granted medRxiv a license to display the preprint in perpetuity.
It is made available under a CC-BY 4.0 International license .

Serologically positive individuals were equally distributed across ages and genders, although the oldest male included in the analysis was also serologically positive (Figure 2).

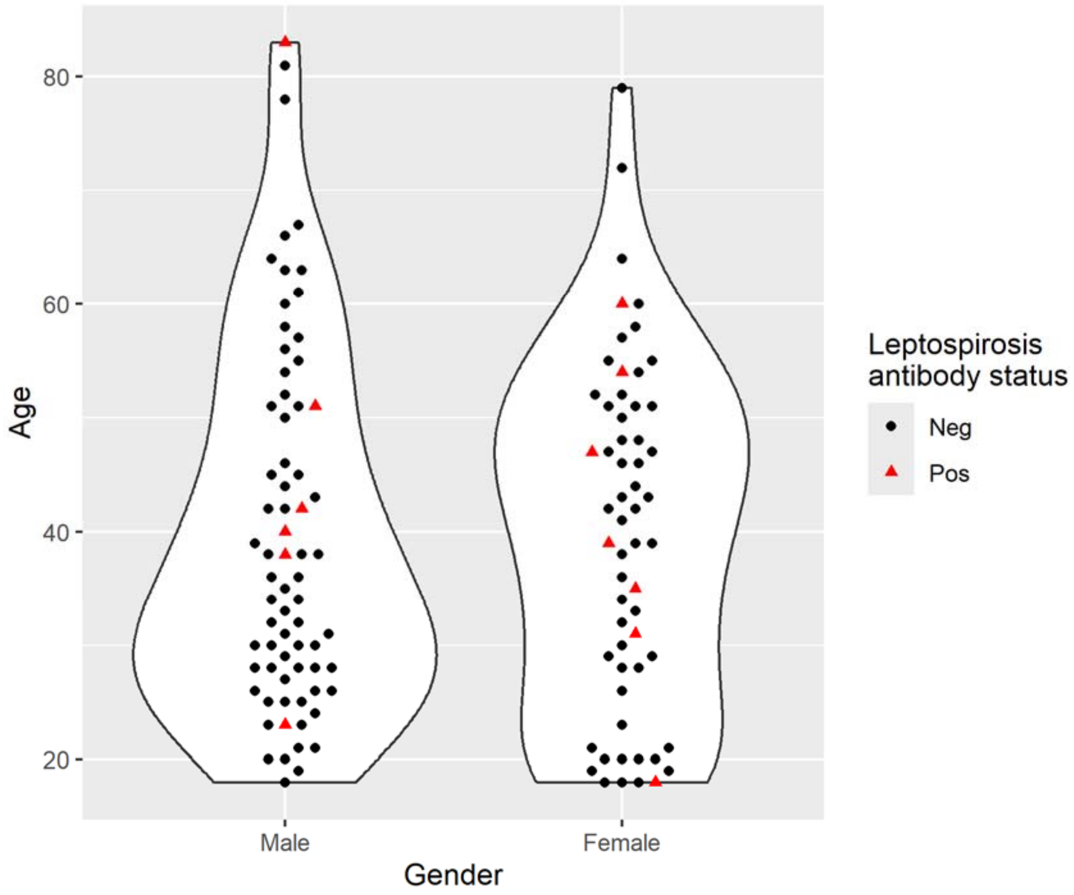

[Figure 2 – Distribution of Leptospirosis antibody status (serological status) by gender and age.]

medRxiv preprint doi: https://doi.org/10.1101/2025.04.28.25326582; this version posted September 8, 2025. The copyright holder for this preprint (which was not certified by peer review) is the author/funder, who has granted medRxiv a license to display the preprint in perpetuity.
It is made available under a CC-BY 4.0 International license .

There were also no significant skews in the household characteristics relative to the environmental factors being analyzed (Figure 3).

[FIGURE 3 – Distribution of nearest distance to each of the environmental factors being analysed (central stream, open sewer points and domestic rubbish piles) by serological status and study area. NA represents rest of households in study area that did not take part in movement analysis.]

Laboratory work

All samples were tested using the MAT test, the reference test for serological diagnosis of leptospirosis, as designated by the WHO. The diagnostic panel used included the following serovars:

- *L. kirschneri* serovar Cynopteri strain 3522C
- *L. kirschneri* serovar Grippothyphosa strain Duyster
- *L. interrogans* serovar Canicola strain H. Ultrech
- *L. interrogans* serovar Autumnlais strain Akiyami A
- *L. borgspetersenii* serovar Ballum strain MUS 127
- *L. interrogans* serovar Copenhageni strain Fiocruz L1-130 (locally isolated in 1996)
- *L. interrogans* serovar Copenhageni strain Fiocruz LV3954

medRxiv preprint doi: https://doi.org/10.1101/2025.04.28.25326582; this version posted September 8, 2025. The copyright holder for this preprint (which was not certified by peer review) is the author/funder, who has granted medRxiv a license to display the preprint in perpetuity.
It is made available under a CC-BY 4.0 International license .

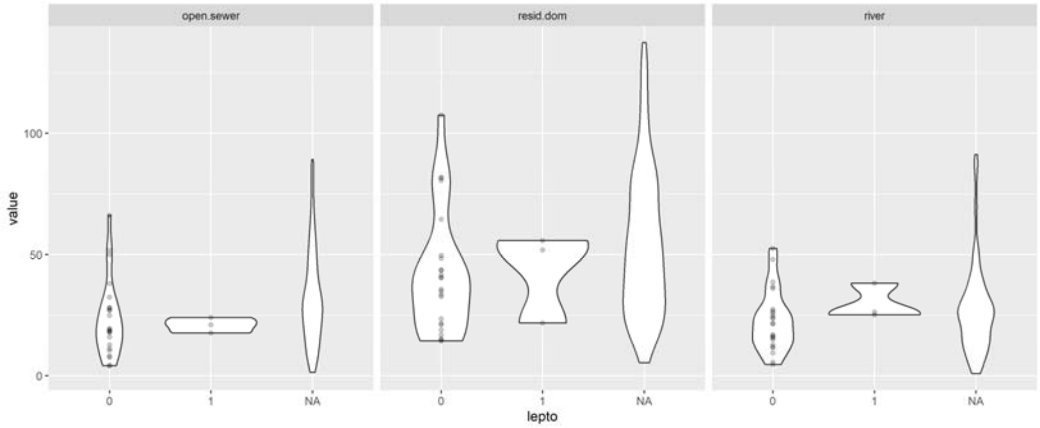

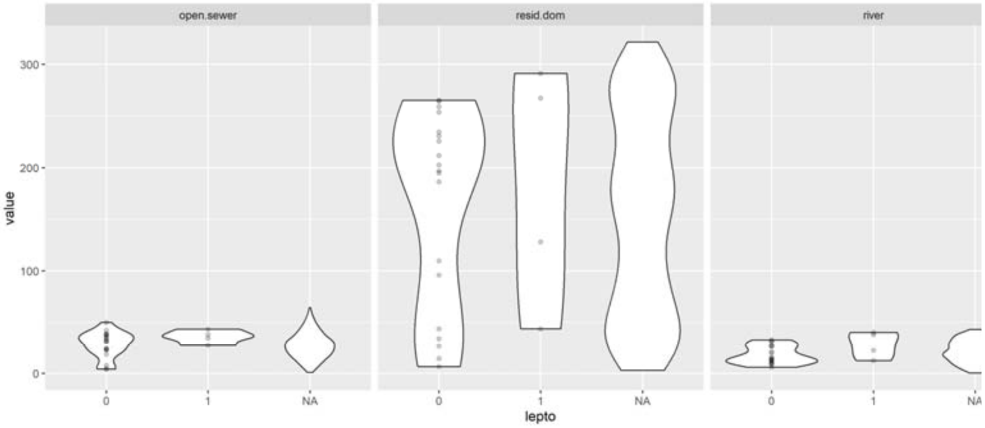

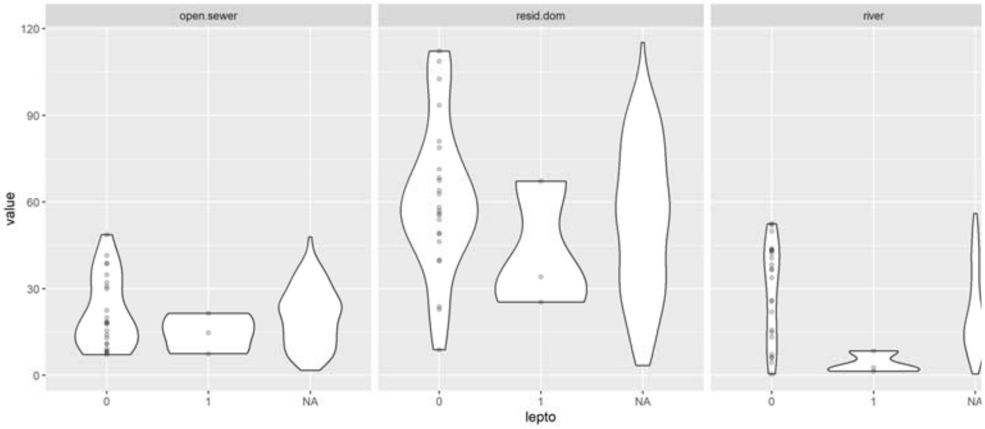

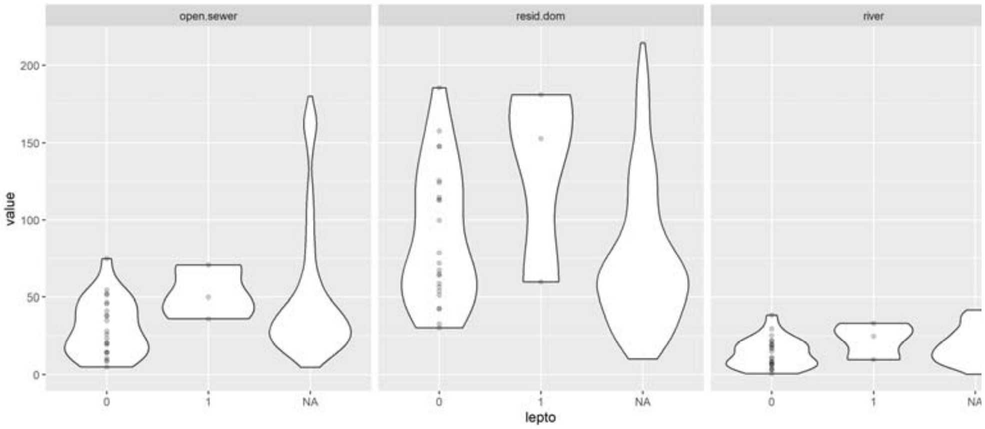

Supplement: 1 [file NIHPP2025.04.28.25326582V3-supplement-1.pdf]
